# Control of Maize Sheath Blight and Elicit Induced Systemic Resistance Using *Paenibacillus polymyxa* Strain SF05

**DOI:** 10.3390/microorganisms10071318

**Published:** 2022-06-29

**Authors:** Bin Chen, Hailiang Han, Junfeng Hou, Fei Bao, Heping Tan, Xiaocheng Lou, Guiyue Wang, Fucheng Zhao

**Affiliations:** Institute of Maize and Featured Upland Crops, Zhejiang Academy of Agricultural Sciences, Dongyang 322100, China; chenbin@zaas.ac.cn (B.C.); hanhl@zaas.ac.cn (H.H.); houjunfeng@zaas.ac.cn (J.H.); baof@zaas.ac.cn (F.B.); tanhp@zaas.ac.cn (H.T.); louxc@zaas.ac.cn (X.L.); wanggy@zaas.ac.cn (G.W.)

**Keywords:** *Paenibacillus polymyxa*, genome sequence, resistance inducement

## Abstract

Maize (*Zea mays* L.) is an important crop in the world and maize sheath blight damages the yield and quality greatly. In this study, an antagonist strain, which exhibited antagonism against pathogenic fungi of maize and controlled maize banded leaf sheath blight in the field, was effectively isolated and named *Paenibacillus polymyxa* strain SF05. High cellulase and chitinase activity of the strain were detected in this study, which might contribute to degrading the cell wall of fungi. Furthermore, different resistant genes such as *ZmPR1a*, *OPR1* and *OPR7* were elicited differently by the strain in the leaves and stems of maize. In order to explain the biocontrol mechanism of *P. polymyxa* strain SF05, the genome was sequenced and then the genes involving the biocontrol mechanism including biofilm formation pathways genes, cell wall degradation enzymes, secondary metabolite biosynthesis gene clusters and volatile organic compounds biosynthesis genes were predicted. The study revealed the biocontrol mechanism of *P. polymyxa* strain SF05 preliminary and laid a foundation for further research of biocontrol mechanism of *P. polymyxa*.

## 1. Introduction

Mazie (*Zae mays* L.) is an important crop in the world. According to FAOSTAT (https://www.fao.org/faostat/en/#home accessed on 17 February 2022), more than 197 million ha of maize were planted all over the world in 2019 and the production reached 1.15 billion tons. The pathogens, including *Bipolaris maydis*, *Exserohilum turcicum*, *Rhizoctonia solani*, *Fusurium verticillioides*, etc., caused great losses of maize in production and quality, and the infected maize even affects human health [1]. Chemical control is the most commonly used method to control maize fungal diseases. Fludioxonil, triazoles, azoxystrobin, etc., are usually used for controlling the diseases caused by the pathogens mentioned above in maize [2,3,4,5]. However, Maize sheath blight caused by *R. solani* is a common disease in maize and it always breaks out in summer and autumn when the maize is nearly ripe [4]. However, maize, especially fresh maize such as waxy and sweet maize, is an important food for human. Meanwhile, the abusive application of chemical pesticides results in problems such as environment pollution [6]. Therefore, chemical pesticides are not suitable to be used too much when maize sheath blight breaks out. In order to control maize sheath blight safely, the demand for safe and clean biological pesticides in production is quite urgent. 

*Paenibacillus polymyxa* is usually reported as plant growth-promoting rhizobacteria (PGPR), which could enhance plant productivity and often elicit plant immunity against multiple plant pathogens [7,8,9]. In previous research, *P. polymyxa* was reported as a safe antagonist for crops and could colonize in the host to fix nitrogen [10,11,12], which was beneficial for plant growth. Antibiotic compounds (polymyxins, fusaricidins, etc.) and hydrolytic enzymes (xylanases, cellulases, pectinases, etc.) secreted by *P. polymyxa* could inhibit the reproduction of pathogens [13]. Furthermore, the expression levels of resistant genes or defense enzymes of plants treated by the bacterium were upregulated, suggesting *P. polymyxa* induced systemic resistance of the host plant [14,15]. However, the molecular mechanism of systemic resistance induced by *P. polymyxa* was still unclear.

The aim of the study is to isolate an antagonistic bacteria strain to control maize sheath blight and determine the biocontrol mechanism of it especially in systemic resistance inducement. In this study, a strain of *P. polymyxa* was isolated from maize and exhibited strong activities to inhibit some pathogenic fungi. Furthermore, different resistant genes were elicited differently by the strain in the leaves and stems of maize. In order to explain the mechanism of induced systemic resistance, the genome of *P. polymyxa* strain SF05 was sequenced and then the genes involving resistance inducing component biosynthesis were predicted. 

## 2. Materials and Methods

### 2.1. Isolation of Antagonistic Bacteria from Maize

The symptomatic sheath tissue was cut into the size of 3 × 3 mm and sterilized with 70% ethanol. The sterilized tissue was placed on the center of potato sucrose agar (PSA) plates at the temperature of 28 °C for two days. Some of the plates showed a zone of inhibition without hyphae, and the bacteria in the center of the zone of inhibition were streaked onto lysogeny broth (LB) agar plants for purification of three generation. To determine the antimicrobial ability of the bacteria, the dish of *Rhizoctonia solani* with a diameter of 5 mm was placed on the center of PSA plate and then 10 μL of bacterial suspension (≈1 × 10^8^ CFU/mL) was dropped on the plate 2 cm away from the dish. The bacterial strain of which the inhibition zone was biggest was selected to continuous culture. Strains that maintained better antibacterial activity after five generations of continuous culture were identified by molecular and biological characteristics.

### 2.2. Identification of Antagonistic Bacteria SF05

Antagonistic bacteria SF05 was isolated from the sheath of maize and exhibited steady and strong antimicrobial activity. The genome DNA of antagonistic bacteria SF05 was extracted by TIANamp Bacteria DNA Kit (DP302-02, TIANGEN, Beijing, China) and 16s rDNA was amplified with the primers: 5′-AGAGTTTGATCATGGCTCAG-3′ (*16s*F) and 5′-TAGGGTTACCTTGTTACGACTT-3′ (*16s*R) [16]. The PCR products were sequenced by Tsingke Biotechnology Co., Ltd. and the sequences were aligned on NCBI with the BLASTN program. The phylogenetic tree of 16S rDNA sequences of SF05 and kindred strains were constructed by MEGA 7.0 with the method of neighbor joining setting the bootstrap as 1000.

The biological characteristics of SF05 were processed by API 20 E: Identification system for Enterobacteriaceae and other non-fastidious Gram-negative rods and BIOLOG system. 

### 2.3. Antimicrobial Activity of Antagonistic Bacteria Strain SF05 to Pathogenic Fungi

To determine the antimicrobial ability of the bacteria, the dish of pathogen fungi with a diameter of 5 mm was placed onto the center of a PSA plate and then 10 μL of bacterial suspension (≈1 × 10^8^ CFU/mL) was dropped onto the plate 2 cm away from the dish. Sterile water was used as negative control (NT). All plates were incubated in 28 °C until the colony of NT was just full of the plates. Pathogenic fungi used in this study are listed in Table 1. The inhibition rate was calculated as: Inhibition rate (%) = (diameter of NT fungi colony − diameter of treated fungi colony)/(diameter of NT fungi colony − diameter of fungi dish) × 100.

### 2.4. Control Effect of P. polymyxa Strain SF05 on Banded Leaf and Sheath Blight in Field

The third sheath of maize (cv. Zhetian 19) at the booting stage (V10) was inoculated by sorghum grains that had been affected by *R. solani*. The bacterial suspension (≈1 × 10^8^ CFU/mL) and sterile water were mixed in the ratio of 1:1 and sprayed on the inoculated sheath of maize at the tassel stage (VT). Armistar Top (Syngenta) diluent (effective constituent: 0.2 g/L) was used as positive control and sterile water and blank LB liquid medium as NT. The disease index of maize banded leaf and sheath blight before treatment and at the milk stage (R4) was investigated by the method described in the Technical Specification on Evaluation of Maize Resistance to Pests—Part 9: Banded Leaf and Sheath Blight. Control effect was calculated as: Control effect (%) = ((disease index of NT − disease index of treated fungi colony)/ disease index of NT) × 100.

### 2.5. Detection of Expression Levels of Resistant Gene Induced by P. polymyxa Strain SF05 in Maize Seedling

Maize seedlings (cv. Zhetian 19) at the V3 stage were sprayed by bacterial suspension (≈1 × 10^8^ CFU/mL) until droplets covered the surfaces of the leaves and stems and incubation was carried out with HiScript III 1st Strand cDNA Synthesis Kit (+gDNA wiper) (R312-01, Vazyme, Nanjing, China). The expression of resistant genes was analyzed using the primers list in Table 2 [17,18,19]. The concentration of cDNA was adjusted to 100 ng/μL. qRT-PCR was conducted using ABI QuantStudio 7 (Thermo Fisher, Waltham, MA, USA). The reaction mixture (5 μL) contained 2.5 μL of 2 × ChamQ Universal SYBR qPCR Master Mix, 0.1 μL of forward primer, 0.1 μL of reverse primers, 1 μL of cDNA and 1.3 μL of ddH_2_O. The thermocycle parameters were as follows: initial polymerase activation for 30 seconds (s) at 95 °C, and then 40 cycles of 30 at 95 °C, 60 s at 58 °C, and 30 s at 72 °C. Relative expression levels were calibrated and normalized to the level of *Actin*. inoculated seedlings in 28 °C greenhouses. Blank LB liquid medium was used as control. Twenty-four hours after inoculation, the total RNA of leaves, stems and roots was extracted by TIANGEN RNAprep Pure Plant Kit (DP432) and then first-strand cDNA synthesis.

### 2.6. Cell Wall Degradation Enzyme Activities of P. polymyxa Strain SF05

Chitinase, pectinase and cellulase were considered as main cell wall degradation enzymes in this study. The activities of cellulase and pectinase were determined according to the methods described by Chen et al. [20]. Chitinase Activity Detection Kit (BC0825, Solarbio, Beijing, China) was used to determine the chitinase activity of *P. polymyxa* strain SF05. Another antagonistic bacterial strain *Bacillus subtilis* BS0512 (Laboratory Collection) and a pathogenic bacterial strain *Dickeya zeae* NCPPB 3531 (Nanjing Agricultural University) were also determined as control.

### 2.7. Genome Sequencing of P. polymyxa Strain SF05

Genomic DNA was extracted with the SDS method [21]. The harvested DNA was detected by the agarose gel electrophoresis and quantified by Qubit^®^ 2.0 (Invitrogen, Thermo Fisher Scientific, Hillsboro, OR, USA). The whole genome of *P. polymyxa* strain SF05 was sequenced using the Nanopore PromethION platform and Illumina NovaSeq PE150 at the Beijing Novogene Bioinformatics Technology Co., Ltd. (Beijing, China). Unicycler was used to combine PE150 data and Nanopore data which were used to assemble then compare the readings to the assembled sequence, count the distribution of sequencing depth, and distinguish whether the assembled sequence was a chromosomal sequence or a plasmid sequence according to sequence length and alignment, and check whether it was a circular genome. The genes of the genome were predicted by the Pan-genome analysis pipeline (PAGP) [22]. 

### 2.8. Prediction of Genes Involving Resistance Inducing Component Biosynthesis of P. polymyxa Strain SF05

Nine *P. polymyxa* genomes (ZF129, CF05, YC0573, Sb3-1, HY96-2, CJX518, JE201, EBL06 and E681) were used in this study to compare with SF05. dbCAN2 meta server (https://bcb.unl.edu/dbCAN2/, accessed on 3 October 2021) was used for the annotation of CWDEs and signal peptide (SP) in *P. polymyxa* genome. Proteins that were both positive in CWDE and SP were considered as the enzymes that could be secreted outside of the cell. The genes involving biofilm formation of selected *P. polymyxa* genomes were predicted by the database of KEGG. Secondary metabolite biosynthetic gene clusters in *P. polymyxa* were analyzed by antiSMASH (https://antismash.secondarymetabolites.org/, accessed on 7 June 2021). According to the method described in Shi [23] and Lee [24], genes involving VOCs biosynthesis were predicted by aligning the protein sequences of SF05 to the database of reported genes from KEGG by the program BLASTP. 

### 2.9. Statistical Analysis

Analysis of variance of experimental data was performed using SPSS Statistics 25 (IBM, Armonk, NY, USA). Significant treatment effects were determined based on the magnitude of the F value (*p* = 0.05). All tests were conducted in triplicate.

## 3. Results

### 3.1. Isolate and Identification of Antagonist Bacterium Strain SF05

**Figure 1:**
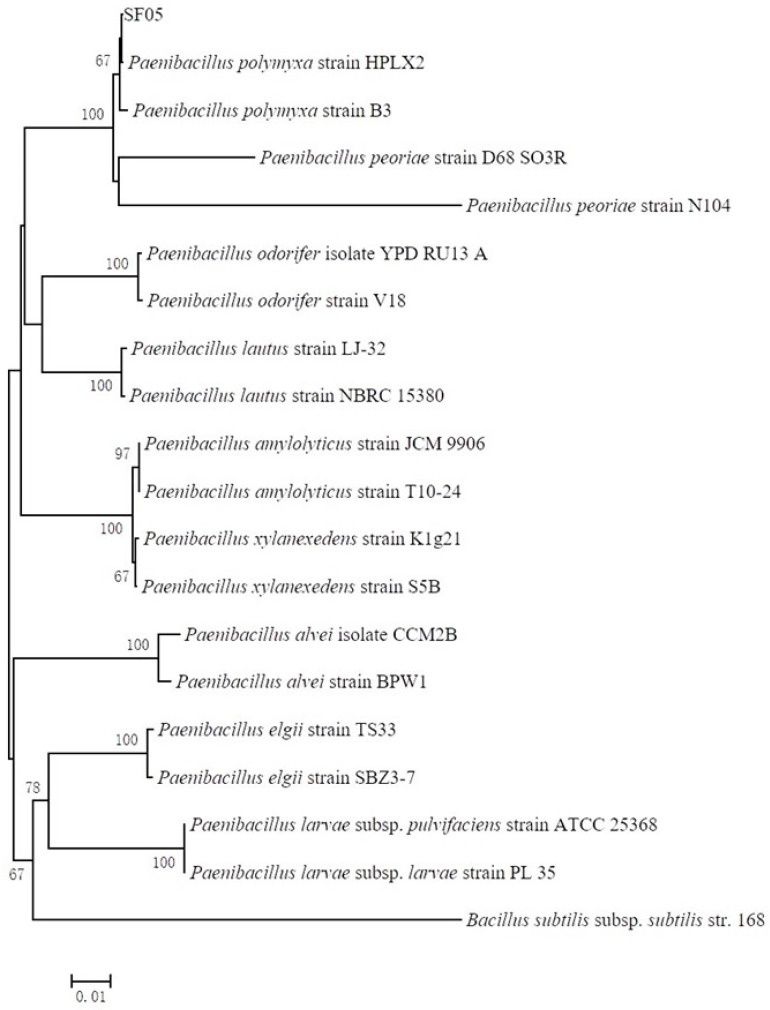
Phylogenetic tree constructed based on sequences of 16S rDNA of *Paenibacillus* genus. The phylogenetic tree was constructed by the neighbor joining method and the sequence of 16S rDNA of *Bacillus subtilis* subsp. *subtilis* str. 168 was used as root. Numbers nearby the branches indicate the percentage of support for 1000 bootstrap resampling analysis. The length of 0.01 was the genetic distance.

After continuous culture for purification, a strain with steady and strong antimicrobial activity was isolated and named SF05 (CCTCC NO: M2020384). The sequence of 16S rDNA was amplified by the primers of 16sF/R and was uploaded to the National Center for Biotechnology Information (NCBI, https://www.ncbi.nlm.nih.gov/, accessed on 3 October 2021) (GenBank: MT820612.1). The alignment results showed that the identities of 16S rDNA and *P. polymyxa* reached 99%, and strain SF05 was in one branch in the phylogenetic tree based on the sequences of 16S rDNA of the *Paenibacillus* genus (Figure 1). The results suggested that antagonist bacterium strain SF05 belonged to *Paenibacillus* genus and might be *P. polymyxa*.

Biological characteristics of SF05 determined by SPI 20 E showed that *P. polymyxa* strain SF05 was positive in β-Galactosidase and gelatinase activities but negative in urease activity (Table S1). The strain could uptake glycerol, ribose, D-xylose, galactose, mannose, glucose, fructose, etc., to produce acid (Table S2). Identification by the BIOLOG system showed that SF05 could assimilate dextrin, D-maltose, D-trehalose, D-cellobiose, gentian disaccharide, sucrose, D-turanose, stachyose, etc., for growth (Table S3). Meanwhile, *P. polymyxa* strain SF05 was observed to grow in the environment of pH = 6 but not in 8% NaCl. Finally, the strain was identified as *P. polymyxa* according to its physiological and biochemical characteristics by Handbook of Systematic for Common Bacteria [25].

### 3.2. Antimicrobial Activity of P. polymyxa Strain SF05 to Pathogenic Fungi

**Figure 2:**
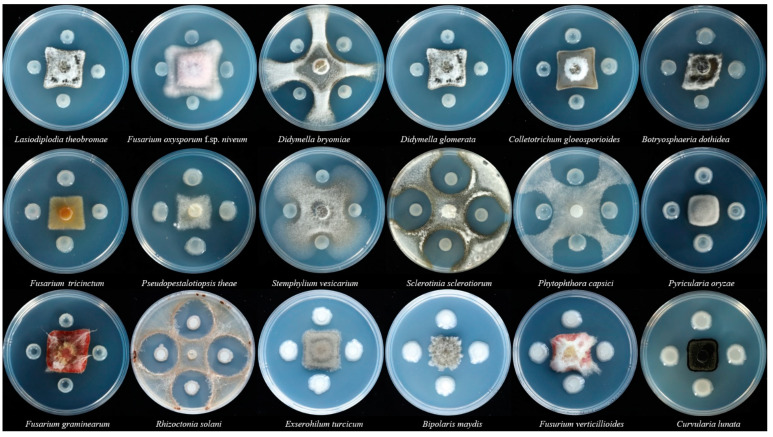
Antagonistic activities of *P. polymyxa* strain SF05 against pathogenic fungi. The antagonistic results were observed enough times after co-inoculation of *P. polymyxa* strain SF05 and pathogenic fungi on PSA plates and incubation at the temperature of 28 °C.

Two to seven days after incubation, *P. polymyxa* strain SF05 exhibited strong antimicrobial activity to most pathogenic fungi tested in this study (Figure 2). The inhibition rates of fungi ranged from 57.06% to 80%. *P. polymyxa* strain SF05 could inhibit some important pathogenic fungi including *P. oryzae*, *S. sclertiorum*, *F. graminearum*, *R. solani*, *E. turcicum*, *B. maydis*, *F. verticillioides*, *C. lunata* which caused great losses in the production of rice, maize, rape and wheat. 

### 3.3. Control Effect of P. polymyxa Strain SF05 on Banded Leaf and Sheath Blight in Field

No symptoms of banded leaf and sheath blight were observed in the field before being treated (data not shown). Thirty-one days later, the disease index of biological and chemical treatment to banded leaf and sheath blight in the field was significantly decreased compared to NT. The control effect of biological control was slightly lower than that of chemical treatment (Table 3).

### 3.4. Expression Levels of Resistant Gene Induced by P. polymyxa Strain SF05 in Maize Seedling Differed in Stem and Leaf

**Figure 3:**
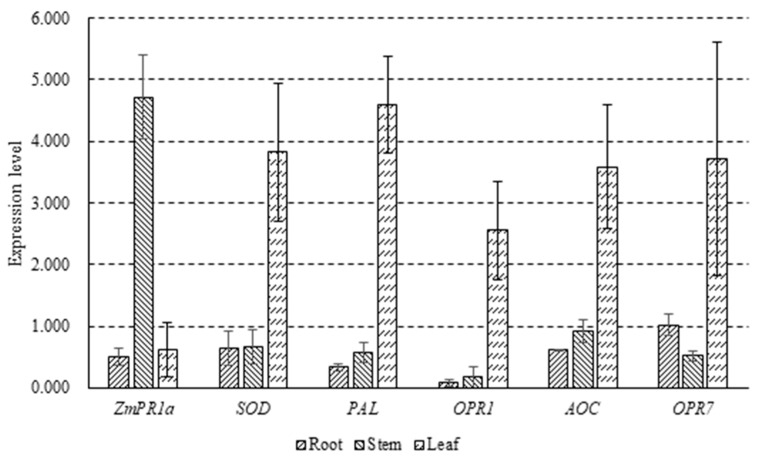
Expression analysis of maize disease resistance genes in different issues after being induced by *P. polymyxa* strain SF05 compared to the control group. The error bars in the figure are mean ± standard deviation.

*OPR1* was downregulated by nearly 92% in root, while the expression levels of most detected genes just changed a little. *ZmPR1a* was upregulated in stem treated by *P. polymyxa* strain SF05 by 4.72-fold and the other genes remained near levels of those in the root. On the contrary, the expression level of *ZmPR1a* did not exhibit an obvious variation in treated leaves to the control but other genes were upregulated by 2.55–4.59-fold (Figure 3).

### 3.5. Cell Wall Degradation Enzyme Activities of P. polymyxa Strain SF05

**Figure 4:**
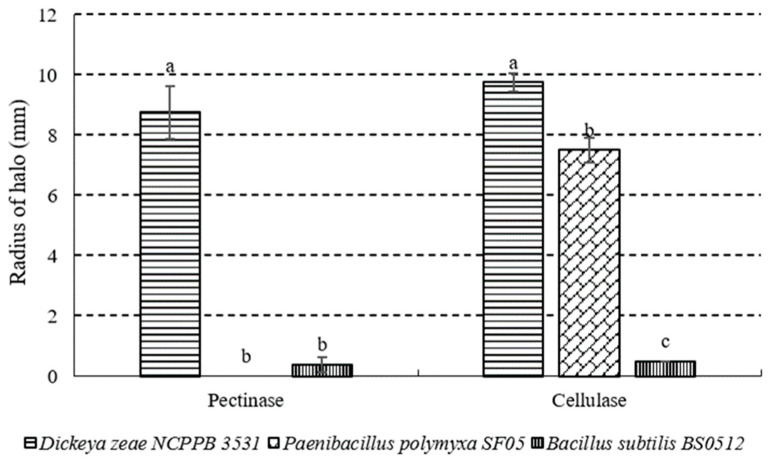
Pectinase and cellulase activities of *D. zeae* NCPPB 3531, *P. polymyxa* strain SF05 and *B. subtilis* BS0512. Different lowercase letters denote significant differences (*p* < 0.05).

**Figure 5:**
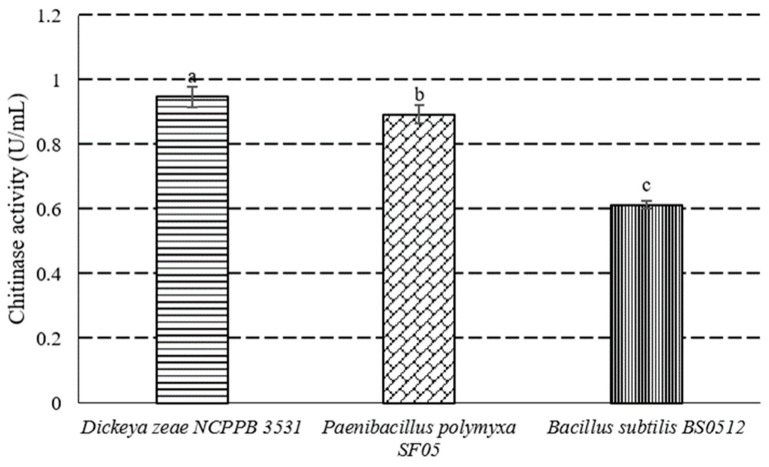
Chitinase activity of *D. zeae* NCPPB 3531, *P. polymyxa* strain SF05, *B. subtilis* BS0512. Different lowercase letters denote significant differences (*p* < 0.05).

The radius of halo degraded by *P. polymyxa* strain SF05 in cellulase activity assay plates was significantly bigger than that by *B. subtilis* BS0512 (Figure 4), which was another antagonist strain. Moreover, the strain did not exhibit any pectinase activity in assay plants. The chitinase activities of three strains tested in this study were sorted from high to low as *D. zeae* NCPPB 3531, *P. polymyxa* strain SF05 and *B. subtilis* BS0512 (Figure 5).

### 3.6. Genome Information of P. polymyxa Strain SF05

The total length of the *P. polymyxa* strain SF05 genome (Accession: CP071875) was 5.46 M with a GC content of 45.5%. No plasmid was found in this genome. A total of 4615 proteins, 43 rRNA and 108 tRNA were annotated in the genome of SF05. Here, nine *P. polymyxa* genomes including the representative genome, ZF129, were selected to compare with the genome of SF05. It was not difficult to find that the genome size and protein numbers of SF05 were less than most genomes (Table 4), suggesting the characteristics might be varied. 

### 3.7. Annotation Information of Biocontrol Mechanism of P. polymyxa Strain SF05

To explore the biocontrol mechanism of *P. polymyxa* strain SF05, biofilm formation pathways genes, cell wall degradation enzymes (CWDEs), secondary metabolite biosynthesis gene clusters and volatile organic compounds (VOCs) biosynthesis genes were predicted and compared with nine other *P. polymyxa* genomes including a representative genome of ZF129. Biofilm formation pathways genes were found in all genomes, suggesting SF05 could form biofilm in maize. In addition, 10 genes were predicted in the pathways of Biofilm formation—*Pseudomonas aeruginosa* in the genome of SF05, which was more than other compared genomes (Table S4). Cellulases, chitinases and peptidoglycan hydrolases were also annotated in 10 genomes of *P. polymyxa* (Table S5) by the CAZy database and 17 related genes were found in the genome of SF05. Secondary metabolite biosynthesis gene clusters in *P. polymyxa* genomes were predicted by antiSMASH (Table S6) and 16 clusters were found in the genome of SF05, in which the Bacitracin biosynthesis gene cluster was only found. Similar to CWDEs, VOCs including 2,3-butanediol biosynthesis genes ranging from 14 to 16 were found in 10 *P. polymyxa* genomes (Table 5). Annotation information revealed that the biocontrol mechanism of *P. polymyxa* strain SF05 was similar to other strains but there were also some differences.

## 4. Discussion

In this study, *P. polymyxa* strain SF05 was isolated and identified which exhibited significant antagonism to some important pathogenic fungi. Moreover, for the first time, it was reported that the *P. polymyxa* strain primed different plant defense genes in different tissues. The genome of the bacterium was sequenced and the expression levels of VOCs biosynthesis genes were analyzed in the cDNA of maize-SF05 interaction to explain the mechanism of biological control. 

Similar with some *Bacillus* and *Paenibacillus* strains, *P. polymyxa* exhibited a strong ability to inhibit the growth of pathogens, which could be used to control some plant disease. It was also reported that *B. velezensis* CE100, *B. amyloliquefaciens* F9 and *B. velezensis* VB7 could be used against *F. oxysporum* effectively [26,27,28]. Likely, *P. polymyxa* strain SF05 could also inhibit the growth of the fungi, suggesting the strain might also help to control the disease caused by *F. oxysporum* in strawberry and banana. In addition, *B. amyloliquefaciens* F9 was also found to be able to control citrus canker caused by *Xanthomonas citri* subsp. *citri* [28]. Therefore, it might be a new insight for *P. polymyxa* strain SF05 to control the bacterial diseases. Different from traditional pre-harvest control, some antagonistic bacteria such as *B. amyloliquefaciens* HF-01 and *P. polymyxa* strain SG-6 were used to control postharvest diseases [29,30], suggesting *P. polymyxa* strain SF05 could be used to control the saprophytic fungi in harvest fruits and crops. 

As was reported, *P. polymyxa* was a PGPR that could colonize plants and inhibit some pathogens. The bacterium was observed colonizing *A. thaliana* and then antagonizing *Pseudomonas syringae* [31], *Phytophthora palmivora* and *Pythium aphanidermatum* [32]. *P. polymyxa* was also isolated from soil around peanut roots and it controlled crown rot disease [33]. Son et al. reported that *P. polymyxa* suppressed the disease complex caused by root-knot nematode and fusarium wilt fungus [9]. It was observed that *P. polymyxa* infected the roots of plants and formed biofilm in intercellular space. Five days after inoculation to roots, more *P. polymyxa* cells were colonized in the leaves of *A. thaliana* rather than in roots, indicating the bacterium could be transferred in the plant [31]. The antagonistic components secreted by *P. polymyxa* were mainly polysaccharides, peptides and proteins, which exhibited superior water solubility, heat resistance and acid-base stability and were easy in store and use [34,35]. The strain isolated and identified in this study exhibited significant antagonism to some important pathogenic fungi, while controlling banded leaf and sheath blight in field effectively, indicating *P. polymyxa* strain SF05 could secret some components to inhibit the fungi and increase the resistance of maize.

Elicitation of ISR is an important part of biological control mechanism of *P. polymyxa*. The phenomenon that the response of *A. thaliana* treated by the bacterium to biotic and abiotic stress increased was firstly reported by Timmusk and Wagner [14]. In addition, gene expression analysis indicated the elicitation of ISR might be related to drought stress caused by *P. polymyxa*. Activities of defense enzymes and content of salicylic acid (SA) in tomato leaves treated by *P. polymyxa* were higher than those in control [15]. Lee et al. and Shi et al. proved that some components secreted by *P. polymyxa* could elicit ISR [23,24]. In this study, *P. polymyxa* strain SF05 induced the expressions of defense genes, which was the same as reported previously. In particular, upregulation of *ZmPR1a* in stem might enhance the resistance to banded leaf and sheath blight in maize [36]. 

Genomic information partly explained the mechanism of antimicrobial activity to pathogen fungi. Biofilms are formed by bacteria that attach to surfaces aggregating in a hydrated polymeric matrix of their own synthesis [37], which could contribute to *P. polymyxa* colonizing the rhizosphere of *Arabidopsis thaliana*, peanut, tomato, cucumber, wheat and maize [33,38,39]. Since the cell wall of fungi and bacterium are mainly composed of cellulose, chitin and peptidoglycan, degradation of the cell wall could inhibit the growth of the pathogen. In addition, secondary metabolite secreted by bacterium usually exhibit antimicrobial activities [40]. Cellulases, chitinases, peptidoglycan hydrolases and secondary metabolite biosynthesis gene clusters were found in the genome, suggesting the bacteria secreted some CWDEs and secondary metabolites that could inhibit the growth of pathogens.

However, *P. polymyxa* strain SF05 primed different plant defense genes in different tissues of maize, which was not reported in previous literature. In order to explain the mechanism preliminarily, the genome of *P. polymyxa* strain SF05 was sequenced. The related annotation information revealed some genes that might be involved in the inducement of resistance. Some VOCs produced by *P. polymyxa* were reported as being able to induce ISR in the host plant [23]. The genes involved in the biosynthesis of the reported VOCs were also found in the genome, suggesting *P. polymyxa* strain SF05 could prime the defense genes by producing VOCs.

## 5. Conclusions

*P. polymyxa* strain SF05 is a potential biological fungicide that could prevent some important diseases while being safe for plants. It was found for the first time that *P. Polymyxa* strain SF05 elicited different resistant genes in leaves and stems of maize. The biocontrol mechanism of *P. polymyxa* strain SF05 might include: (i) inhibiting the growth of pathogens by secreting CWDEs and secondary metabolites; (ii) producing VOCs to induce the defense response of maize. The study laid a foundation for research on the function of VOCs biosynthesis genes and the roles they play in the network of defense gene expression regulation in interaction of maize-SF05.

## Figures and Tables

**Table 1:** Pathogenic fungi used in this study.

Species	Source
*Rhizoctonia solani*	Laboratory Collection
*Exserohilum turcicum*	Laboratory Collection
*Bipolaris maydis*	Laboratory Collection
*Fusurium verticillioides*	Laboratory Collection
*Sclerotinia sclerotiorum*	Laboratory Collection
*Curvularia lunata*	Hebei Academy of Agricultural and Forestry Sciences
*F. graminearum*	Nanjing Agricultural University
*Lasiodiplodia theobromae*	Nanjing Agricultural University
*F. oxysporum* f.sp. *niveum*	Nanjing Agricultural University
*Didymella bryoniae*	Nanjing Agricultural University
*D. glomerata*	Nanjing Agricultural University
*Colletotrichum gloeosporioides*	Nanjing Agricultural University
*Botryosphaeria dothidea*	Nanjing Agricultural University
*F. tricinctum*	Nanjing Agricultural University
*Pseudopestalotiopsis theae*	Nanjing Agricultural University
*Stemphylium vesicarium*	Nanjing Agricultural University
*Phytophthora capsici*	Nanjing Agricultural University
*Pyricularia oryzae*	Nanjing Agricultural University

**Table 2:** Sequences of primers used in qRT-PCR.

Primer	Sequences (5′-3′)	References
*Actin*-F	GGTTCTATTCCAGCCATCCTTCATTG	[12]
*Actin*-R	TCTCCTTGCTCATGCGGTCAC	
*ZmPR1a*-F	GGCGAGAGCCCCTACTAGAC	[13]
*ZmPR1a*-R	AAATCGCCTGCATGGTTTTA	
*PAL*-F	GAAGCTCATGTCGTCCACCTA	[12]
*PAL*-R	GTTCATGGTCAGCACCTTCTT	
*SOD*-F	AGAATAACATCCCGAAGACATC	[12]
*SOD*-R	AGCCAACAGTCCAACACAGT	
*OPR1*-F	CGTATGGGAGGCTGTTCTTG	[12]
*OPR1*-R	AGCGGTCGTATTTGTTGAGTG	
*OPR7*-F	GAGAAAGGTGGTTGATGCTGTT	[12]
*OPR7*-R	GGAGTTGGATACTTGCCATAGG	
*AOC*-F	GGGCATCTGCGTGCTCATC	[14]
*AOC*-R	ACCGCCAGGTACGACTCCTC	

**Table 3:** Control effect of *P. polymyxa* strain SF05 on maize to banded leaf and sheath blight in the field.

Treatment	Disease Index	Control Effect
Biological Treatment	37.38 ± 1.23 b	37.06%
Chemical Treatment	32.20 ± 0.75 a	45.79%
Negative Control	59.40 ± 2.17 c	-

Different lowercase letters denote significant differences (*p* < 0.05).

**Table 4:** Genome information of *P. polymyxa* was analyzed in this study.

Strain	GenBank	Size (Mb)	GC%	Protein	rRNA	tRNA	Other RNA	Gene	Pseudogene
SF05	-	5.46	45.5	4615	43	108	4	5018	248
ZF129	GCA_006274405.1	5.82	45.4	4873	42	111	4	5115	85
CF05	GCA_000785455.1	5.76	45.5	4738	43	107	4	4966	74
YC0573	GCA_001874425.3	6.13	45.6	5091	37	102	4	5442	208
Sb3-1	GCA_000819665.1	5.83	45.5	4812	46	109	4	5097	126
HY96-2	GCA_002893885.1	5.75	45.6	4663	42	110	4	4955	136
CJX518	GCA_014854715.1	5.69	45.4	4749	42	111	4	5034	128
JE201	GCA_019852195.1	6.17	45.3	5145	46	111	4	5425	119
EBL06	GCA_000955925.1	5.68	45.6	4821	7	38	4	4964	94
E681	GCA_014706575.1	5.42	45.8	4532	36	91	4	4872	209

**Table 5:** Prediction of volatile organic compounds biosynthesis genes in *P. polymyxa* genomes.

VOC	1	2	3	4	5	6	7	8	9	10
2,3-Butanediol	2	2	2	2	2	2	2	2	2	2
Acetone	3	3	3	3	3	3	3	3	3	3
Acetylene	1	1	1	1	1	1	1	1	1	1
Glyoxylic acid	1	0	1	1	1	1	1	1	1	1
Benzaldehyde	2	2	2	2	2	2	2	2	2	1
Butanol	1	1	1	1	1	1	1	1	1	1
Ethanol	3	2	3	2	2	2	2	2	2	3
Methanethiol	2	3	3	3	3	3	3	3	3	3
Total	15	14	16	15	15	15	15	15	15	15

The numbers in the first line in the table represented the genome of *P. polymyxa* SF05 (1), ZF129 (2), CF05 (3), YC0573 (4), Sb3-1 (5), HY96-2 (6), CJX518 (7), JE201 (8), EBL06 (9) and E681 (10).

## Data Availability

All relevant data are presented in the article. Raw data can be provided on reasonable request.

## Supplementary Materials

The following supporting information can be downloaded at: https://www.mdpi.com/article/10.3390/microorganisms10071318/s1, Table S1: Physiological and biochemical characteristics (enzyme activities, carbon source oxidation) of *P. polymyxa* strain SF05; Table S2: Physiological and biochemical characteristics (acid production from carbon source) of *P. polymyxa* strain SF05; Table S3: Physiological and biochemical characteristics (BIOLOG carbon assimilation) of *P. polymyxa* strain SF05; Table S4: Prediction of biofilm formation pathways genes in *P. polymyxa* genomes; Table S5: Prediction of cell wall degradation enzyme genes in *P. polymyxa* genomes; Table S6: Prediction of secondary metabolite biosynthesis gene cluster in *P. polymyxa* genomes.
