# Supplementary material for: Control of Maize Sheath Blight and Elicit Induced Systemic Resistance Using Paenibacillus polymyxa Strain SF05
Source: Microorganisms. 2022 Jun 29;10(7):1318. doi: 10.3390/microorganisms10071318 (PMC9322256; doi:10.3390/microorganisms10071318)
Supplement: Supplementary file 1 [file microorganisms-10-01318-s001.zip › microorganisms-1782218-supplementary.pdf]

Table S1 Physiological and biochemical characteristics (enzyme activities, carbon source oxidation) of *P. polymyxa* strain SF05

		Substrate and enzyme	Result
ONPG	Ortho-Nitrophenyl-Galactoside	β- Galactosidase	+
ADH	Arginine	Arginine-Dihydrolase	-
LDC	Lysine	Lysine Decarboxylase	-
ODC	Ornithine	Ornithine Decarboxylase	-
CIT	Sodium Citrate Dihydrate	Citric Acid Utilize	-
H2S	Sodium Thiosulfate	H2S Generate	-
URE	Urea	Urease	-
TDA	Tryptophan	Tryptophan Deaminase	-
IND	Tryptophan	Indole Produce	-
VP	Pyruvate	3-hydroxybutanone to acetyl methyl methanol	+
GEL	Kohn Gelatin	Gelatinase	+
GLU	Glucose	Fermentation / Oxidation（4）	-
MAN	Mannitol	Fermentation / Oxidation（4）	-
INO	Inositol	Fermentation / Oxidation（4）	-
SOR	Sorbitol	Fermentation / Oxidation（4）	-
RHA	Rhamnose	Fermentation / Oxidation（4）	-
SAC	Sucrose	Fermentation / Oxidation（4）	-
MEL	Melibiose	Fermentation / Oxidation（4）	-

AMY	Amygdalin	Fermentation / Oxidation（4）	-
ARA	Arabinose	Fermentation / Oxidation（4）	-

+: Positive；　-: Negative

Table S2 Physiological and biochemical characteristics (acid production from carbon source) of *P. polymyxa* strain SF05.

Substrate	Result	Substrate	Result
0 Contrast	-	25 Esculin Hydrate	+
1 Glycerol	+	26 Salicin	+
2 Erythritol	-	27 Cellobiose	+
3 D-Arabinose	-	28 Maltose	+
4 L-Arabinose	+	29 Lactose	+
5 Ribose	+	30 Melibiose	+
6 D-Xylose	+	31 Sucrose	+
7 L-Xylose	-	32 Trehalose	+
8 Adonitol	-	33 Synanthrin	+
9 β-Methyl-D-Xyloside	-	34 Melezitose	-
10 Galactose	+	35 Raffinose	+
11 Glucose	+	36 Amylum	+
12 Fructose	+	37 Glycogen	+
13 Mannose	+	38 Xylitol	-
14 Sorbitol	-	39 Geraniol	W
15 Rhamnose	-	40 D-Turanose	W
16 Dulcitpl	-	41 D-Lyxose	-
17 Inositol	-	42 D-Tagatose	-

18 Mannitol	+	43 D- Fucose	-
19 Sorbitol	-	44 L- Fucose	-
20 α-Methyl-D- Mannoside	-	45 D-Arabinitol	-
21 α-Methyl-D- Glucoside	+	46 L-Arabinitol	-
22 N-Acetyl-Glucosamine	-	47 Gluconate	-
23 Amygdalin	+	48 2-Keto-Gluconate	-
24 Arbutin	+	49 5-Keto-Gluconate	-

+: Positive； -: Negative; W: Weakly positive

Table S3 Physiological and biochemical characteristics (BIOLOG carbon assimilation) of *P. polymyxa* strain SF05.

Test item		Result	Test item		Result
A1	Negative Control	-	E1	Gelatin	-
A2	Dextrin	+	E2	Glycyl-L-Proline	-
A3	D-Maltose	+	E3	L-Alanine	-
A4	D-Trehalose	+	E4	L-Arginine	-
A5	D-Cellobiose	+	E5	L-Aspartic Acid	-
A6	Gentiobiose	+	E6	L-Glutamic Acid	-
A7	Sucrose	+	E7	L-Histidine	-
A8	D-Turanose	+	E8	L-Pyroglutamic Acid	-
A9	Stachyose	+	E9	L-Serine	-
A10	Positive Control	+	E10	Lincomycin	W
A11	pH6	+	E11	Guanidine HCl	+
A12	pH5	W	E12	Niaproof 4	-
B1	D-Raffinose	+	F1	Pectin	+
B2	α-D-Lactose	+	F2	D-Galacturonic Acid	+
B3	D-Melibiose	+	F3	L-Galactonic-Acid Lactone	+
B4	β-Methyl-D-Glucoside	+	F4	D-Gluconic Acid	+
B5	D-Salicin	+	F5	D-Glucuronic Acid	-
B6	N-Acetyl-D-Glucosamine	-	F6	Glucuronamide	-

B7	N-Acetyl-β-D-Mannosamine	-	F7	Mucic Acid	-
B8	N-Acetyl-D-Galactosamine	-	F8	Quinic Acid	-
B9	N-Acetyl Neuraminic Acid	-	F9	D-Saccharic Acid	-
B10	1% NaCl	+	F10	Vancomycin	-
B11	4% NaCl	W	F11	Tetrazolium Violet	W
B12	8% NaCl	-	F12	Tetrazolium Blue	W
C1	α-D-Glucose	+	G1	p-Hydroxy-Phenylacetic Acid	-
C2	D-Mannose	+	G2	Methyl Pyruvate	-
C3	D-Fructose	+	G3	D-Lactic Acid Methyl Ester	-
C4	D-Galactose	+	G4	L-Lactic Acid	-
C5	3-Methyl-Glucose	-	G5	Citric Acid	-
C6	D-Fucose	-	G6	α-Keto-Glutaric Acid	-
C7	L-Fucose	-	G7	D-Malic Acid	-
C8	L-Rhamnose	-	G8	L-Malic Acid	-
C9	Inosine	-	G9	Bromo-Succinic Acid	-
C10	1% Sodium Lactate	+	G10	Nalidixic Acid	-
C11	Fusidic Acid	-	G11	Lithium Chloride	+
C12	D-Serine	-	G12	Potassium Tellurite	+
D1	D-Sorbitol	-	H1	Tween 40	-
D2	D-Mannitol	+	H2	γ－Amino-Butyric Acid	-
D3	D-Arabinol	-	H3	α-Hydroxy-Butyric Acid	-

D4	myo-Inositol	-	H4	β-Hydroxy-D,L Butyric Acid	-
D5	Glycerol	+	H5	α-Keto-Butyric Acid	-
D6	D-Glucose-6-PO4	-	H6	Acetoacetic Acid	-
D7	D-Fructose-6-PO4	-	H7	Propionic Acid	-
D8	D- Aspartic Acid	-	H8	Acetic Acid	-
D9	D-Serine	-	H9	Formic Acid	-
D10	Troleandomycin	-	H10	Aztreonam	-
D11	Rifamycin SV	-	H11	Sodium Butyrate	+
D12	Minocycline	-	H12	Sodium Bromate	W

+: Positive； -: Negative; W: Weakly positive.

Table S4 Prediction of biofilm formation pathways genes in *P. polymyxa* genomes.

**Pathway**	**1**	**2**	**3**	**4**	**5**	**6**	**7**	**8**	**9**	**10**
**Biofilm formation – *Escherichia coli***	13	13	14	14	12	13	14	13	14	14
**Biofilm formation – *Pseudomonas aeruginosa***	10	9	9	9	8	9	9	9	9	8
**Biofilm formation – *Vibrio cholerae***	12	11	12	12	11	11	11	11	12	12
**Total**	35	33	35	35	31	33	34	33	35	34

*: The numbers in the first line in the table represented the genome of *P. polymyxa* SF05 (1), ZF129 (2), CF05 (3), YC0573 (4), Sb3-1 (5), HY96-2 (6), CJX518 (7), JE201 (8), EBL06 (9) and E681 (10).

Table S5 Prediction of cell wall degradation enzyme genes in *P. polymyxa* genomes.

Family	Function	1	2	3	4	5	6	7	8	9	10
**CBM16**		1	1	1	0	1	1	1	1	1	0
**CBM46**		1	2	2	1	2	2	2	2	2	2
**CBM59**	Cellulose degradation	0	1	1	0	0	1	1	1	1	0
**CBM63**		1	1	1	1	1	1	1	1	1	1
**GH5**		4	6	6	4	6	6	7	6	6	5
**CBM3**		2	5	5	5	5	5	5	5	5	5
**CBM50**		1	1	1	1	1	1	1	1	1	0
**CBM54**		0	0	0	0	0	0	1	0	0	0
	Chitin degradation										
**GH16**		2	1	1	1	2	1	2	1	1	0
**GH18**		3	2	3	2	3	3	3	3	3	3
**GH48**		0	1	1	1	1	1	1	1	1	1
**PL9**	Peptidoglycan degradation	2	2	2	2	2	2	2	2	2	2
**Total**		17	23	24	18	24	24	27	24	24	19

*: The numbers in the first line in the table represented the genome of *P. polymyxa* SF05 (1), ZF129 (2), CF05 (3), YC0573 (4), Sb3-1 (5), HY96-2 (6), CJX518 (7), JE201 (8), EBL06 (9) and E681 (10).

Table S6. Prediction of secondary metabolite biosynthesis gene cluster in *P. polymyxa* genomes.

Cluster	1	2	3	4	5	6	7	8	9	10
**Nostamide A**	0	1	1	0	0	1	0	1	0	0
**Aurantinin**	0	1	1	1	1	1	1	1	1	1
**Bacitracin**	1	0	0	0	0	0	0	0	0	0
**Bogorol A**	0	0	0	0	1	0	0	0	0	0
**Brevicidine**	0	0	1	1	1	0	0	1	0	0
**Fusaricidin B**	1	1	1	1	1	1	1	1	1	1
**Lipopolysaccharide**	0	0	0	0	0	0	0	0	1	1
**Marthiapeptide A**	0	0	1	0	0	1	0	0	0	0
**Octapeptin C4**	0	0	0	1	0	0	0	0	0	0
**Paenibacillin**	1	1	0	0	0	0	0	0	0	0
**Paenibacterin**	0	1	0	0	0	1	1	0	0	0
**Paenicidin A**	0	1	0	0	0	1	0	0	0	0
**Paenicidin B**	1	0	1	0	0	0	0	1	0	0
**Paenilan**	0	1	1	1	1	1	1	1	1	1
**Paenilipoheptin**	2	0	0	2	1	0	1	0	1	1
**Paeninodin**	1	1	1	1	0	1	0	1	1	1
**Polymyxin**	0	1	0	0	0	1	0	1	1	1
**Polymyxin B**	1	0	0	0	1	0	1	0	0	0
**Siphonazole**	0	0	0	0	0	0	0	1	0	0

**S-Layer Glycan**	0	0	1	0	1	1	1	1	0	0
**Tauramamide**	1	0	0	1	0	0	0	0	0	0
**Tridecaptin**	1	1	1	1	1	1	1	2	1	1

*: The numbers in the first line in the table represented the genome of *P. polymyxa* SF05 (1), ZF129 (2), CF05 (3), YC0573 (4), Sb3-1 (5), HY96-2 (6), CJX518 (7), JE201 (8), EBL06 (9) and E681 (10).
